# Analysis of nascent silicon phase-change gratings induced by femtosecond laser irradiation in vacuum

**DOI:** 10.1038/s41598-018-30269-0

**Published:** 2018-08-21

**Authors:** Felice Gesuele, Jijil JJ Nivas, Rosalba Fittipaldi, Carlo Altucci, Riccardo Bruzzese, Pasqualino Maddalena, Salvatore Amoruso

**Affiliations:** 1 Dipartimento di Fisica “Ettore Pancini”, Università di Napoli Federico II, Complesso Universitario di Monte S. Angelo, Via Cintia, I-80126 Napoli, Italy; 2 CNR-SPIN UOS Napoli, Complesso Universitario di Monte S. Angelo, Via Cintia, I-80126 Napoli, Italy; 3 CNR-SPIN, UOS Salerno, Via Giovanni Paolo II 132, I-84084 Fisciano, Italy

## Abstract

The formation of periodic surface structures is a general effect of femtosecond laser irradiation of solid targets showing promising interest in material science and technology. However, the experiments are typically carried out in air, a condition in which the target surface becomes densely decorated with nanoparticles that can influence the formation of the surface structures in the early stage of the irradiation process. Here we report an investigation of structures generation on a silicon surface irradiated in vacuum (10^−5^ mbar) with a low number of laser pulses (*N* ≤ 10) that exploits several microscopy techniques (optical, atomic force, electron and Raman). Our analyses allow identifying the creation of silicon phase-change gratings consisting of alternating amorphous and crystalline periodic lines, with almost no material removal, located at the periphery of a shallow ablation crater. These gratings originate from two different kinds of defects: (i) the first is characterized by a peculiar lobed shape that is produced by the first few laser pulses; (ii) the second is provided by the one-dimensional, linear singularity defined by the ablation edge of the nascent crater. Both kind of defects lead to grating structures extending outwards the amorphous central area of the crater along the direction of the laser polarization. Comparative analysis with the surface formed in air, in the same experimental conditions, evidences the important role played by nanoparticles densely decorating the target in air and the striking variation occurring in vacuum.

## Introduction

Direct femtosecond (fs) laser surface structuring is a versatile method to tailor material properties and the formation of laser-induced periodic surface structures (LIPSS) is a striking and extensively studied phenomenon with several applications^1–3^. There is an ongoing debate about LIPSS generation mechanisms, which include inhomogeneous energy deposition, self-organization of surface instabilities, hydrodynamics, etc.^3–7^. However, the most established picture considers that interference between incident laser light and surface waves^3–7^, which can be either light scattered at a rough surface or surface plasmon polaritons (SPPs), causes a modulation of the light intensity distribution that is eventually imprinted onto the material surface. The generation of LIPSS with a spatial period smaller than the laser wavelength by fs laser irradiation is observed in a broad range of materials, including metals, semiconductors and dielectrics. These LIPSS are generally dubbed as *ripples* and in metals and semiconductors are preferentially oriented along the direction normal to laser polarization^3,8^.

Most studies on ripples formation have shown that they are namely generated in ablation conditions induced either by exploiting multi-pulse irradiation (typically a number of pulses *N* ≥ 10, e.g.) with fluence near the single-pulse ablation threshold or few pulses with fluence larger than single-pulse ablation threshold^3,7,9–11^. LIPSS creation is challenging as it can involve a complex series of processes and feedback mechanisms. Generally, the first pulse only produces the creation of some surface defects and decoration with nanoparticles, which may subsequently act as scattering centers for the generation of surface waves in the next pulses. At a slightly larger number of pulses (2 < *N* < 10, e.g.), rudiments of more ordered surface structures start forming that eventually lead to ripples^3,9,12^.

Due to its great technological importance, silicon is one of the most investigated material. Although extensive work has been carried out with respect to the dependence of the ripples characteristics on fluence and number of laser pulses hitting a silicon target surface, less work has been devoted to a detailed analysis of the ripples formation in vacuum^13–15^. However, some of these studies are limited to ripples formation induced by several hundred or a thousand of laser pulses^13,15^. Here we report an analysis of the surface of an intrinsic (100) silicon target after irradiation with a low number (*N* ≤ 10) of fs laser pulses, in vacuum (<10^-5^ mbar). Reduced background pressure limits back-deposition of nanoparticles and its direct influence on surface structuring and may allow gaining further information on the process. The laser source is a Nd:glass laser system that delivers linearly polarized pulses with a Gaussian spatial profile at a repetition rate of 33 Hz. The pulse duration and wavelength are estimated, by means of temporal and spectral characterization^16^, to be ≈850 fs and 1055 nm respectively (see Methods). A detailed characterization of the surface structures is achieved by exploiting several microscopy techniques (optical, atomic force, electron and Raman). Besides the ripples, other LIPSS are observed at the periphery of the ablation crater that consist of alternating amorphous and crystalline periodic stripes, with almost no material removal. The origin of these stripes can be ascribed to a modulation of the deposited energy in the beam peripheral regions as a consequence of surface-scattered electromagnetic waves (SSW) interfering with the low-energy tail of the Gaussian beam. The source of the SSW is identified in defects like the edge of the nascent ablation crater and some surface structure with a peculiar lobed shape formed in the first few pulses. The local absorbed fluence is not enough to induce ablative removal of target material but its spatial modulation is capable to induce local modifications of the near-surface material phase, which are clearly recognized as localized surface fringe patterns by optical, Raman and electron microscopies. With further irradiation, such surface structures likely influence the subsequent increase of the modified surface area typically observed in direct fs laser surface structuring. In fact, the rippled region progressively extend outwards the crater formed by previous pulses in direction of the laser polarization at increasing number of pulses. It is worth noticing that the formation of periodic patterns of amorphous and crystalline regions was recently reported during few pulses fs laser irradiation of p-doped (100) silicon^17^. Moreover, extension of this fringe pattern over larger areas and period tuning by appropriate selection of the experimental conditions have also been recently demonstrated^18,19^. Differently from the present investigation, in the previous cases the fringe pattern forms in air at the center of the irradiation spot in sub-ablative experimental conditions. We have recently addressed a key influence of the environment by studying LIPSS formation for pressure going from ≈10^−5^ mbar to atmospheric conditions^15^. Here, we evidence the formation of silicon phase-change gratings in vacuum, which are very seldom investigated. Our analyses convincingly address the role of laser-induced defects on the formation of nascent ordered structures that can subsequently influence the successive dynamics of the laser surface structuring process.

## Results

### Surface structures generated in vacuum

**Figure 1:**
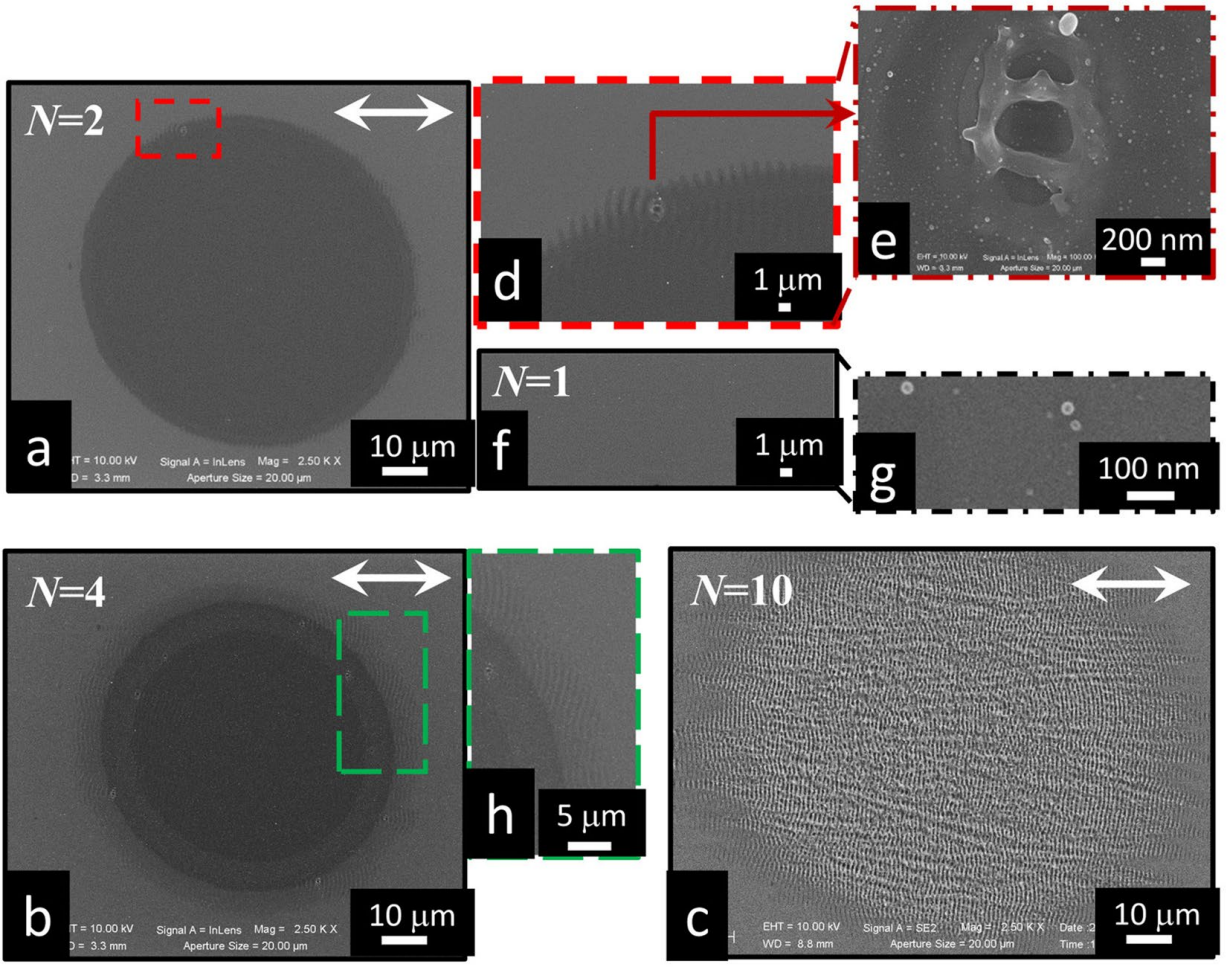
Exemplificative set of SEM images illustrating the surface morphology developed at different number of laser pulses *N*, in vacuum. Panels (**a**) and (**b**) report images for *N* = 2 and *N* = 4 pulses, respectively, registered with the IL detector. Panel **(c)** shows an image for *N* = 10 pulses registered with the ET detector. Inset (**d**) is a zoomed view of the area indicated by the red dashed box in panel (a) showing a defect surrounded by angular fringes, while inset (**e**) is an image of this defect acquired at large magnification. Panel (**f**) reports a SEM image registered in the surface region of the sample irradiated by a single (*N* = 1) laser pulse, while inset (**g**) shows a magnified view of an area in the same region decorated with nanoparticles. Inset (**h**) displays a portion of panel (b) with higher magnification. The double-headed arrow indicates the polarization of the incident laser pulses.

Figure 1 reports an illustrative set of SEM micrographs of the silicon surface after irradiation with different number of laser pulses, namely *N* = 1, 2, 4 and 10, at an energy *E*_0_ = 140 μJ, in vacuum. The corresponding average fluence and intensity are *F* = 0.26 J/cm^2^ and *I* = 3 × 10^11^ W/cm^2^, respectively. In particular, panels (a), (b) and (c) of Fig. 1 show the surface modifications produced after a sequence of *N* = 2, 4 and 10 laser pulses, respectively. It is worth noticing that SEM images of panels (a) and (b) are acquired with the In-Lens (IL) detector, since SEM analysis with the Everhart-Thornley (ET) detector on the same spots is not able to evidence significant morphological changes. Instead, the SEM image of Fig. 1(c) is acquired with the ET detector and features the formation of near-wavelength ripples on the sample surface after irradiation with many laser pulses (e.g. *N* = 10).

As can be seen in the SEM image of Fig. 1(a), a sequence of two pulses induces a clear change producing a smooth and almost featureless surface morphology that can be ascribed to amorphization of the silicon target surface^20^. Moreover, localized wavelike fringes are observed in some location at the spot edge around small surface defects, an example of which is shown in panel (d) of Fig. 1. Figure 1(e) reports a zoomed view of the defect that illustrates its peculiar shape. As a comparison, panels (f) and (g) of Fig. 1 report a SEM image of a region of the sample surface irradiated by a single pulse (*N* = 1) displaying decoration with dispersed nanoparticles and a negligible degree of morphological modifications. Hence, our experimental conditions correspond to a laser fluence below the single pulse ablation threshold. The formation of circular defects on silicon irradiated by a single pulse in air at laser fluences larger than 1 J/cm^2^ have been addressed in some papers^11,20^. Concentric periodic structures around circular defects were reported by Derrien *et al*.^11^ and ascribed to SPPs excitation. For single pulse irradiation the period of such structures is larger than the laser wavelength and reduces to values below the laser wavelength for few pulses (2 ≤ *N* ≤ 4). Bonse *et al*.^20^ observed the formation of circular holes with diameters in the range (1–6) μm and depths up to about 100 nm within the crater. These holes were interpreted as resulting from the formation of bubble in the liquid layer induced by the laser irradiation and vanished or were obscured by other surface structures by subsequent pulses. Varlamova *et al*.^9,21^ observed the formation of a concentric pattern of regular sub-wavelength ripples after single shot during irradiation of silicon in ultra-high vacuum (10^−9^ mbar) at about the ablation threshold (0.215 J/cm^2^). The ripples orientation followed the shape of the irradiated spot without any reference to the direction of the linear laser polarization. Moreover, several perfectly round conical holes with diameter comparable to ripples spacing and depth of about 50 nm decorated the irradiated spot. In our case, single pulse irradiation induces a negligible degree of morphological changes, but defects with an unusual shape are formed after the next few pulses. In fact, the defect shape is characterized by an approximately elliptical contour with the minor axis of ≈1.0 μm, directed along the laser polarization, and the major axis of ≈1.6 μm (see Fig. 1(e)). Moreover, it presents two ridges with a thickness of ≈200 nm directed along the minor axis separating the internal area in three parts. The observed fringes (see Fig. 1(d)) seem to depart from the local defect and propagate along the direction of the laser polarization.

As an example of the intermediate surface state preceding the formation of well-developed ripples, Fig. 1(b) reports a SEM image corresponding to *N* = 4. One can appreciate a further change of the surface morphology with respect to *N* = 2. In particular, three different regions are clearly identified that are likely associated to the Gaussian spatial profile of the laser pulse fluence. A central, circular region with a diameter of ≈50 μm is surrounded by an annular strip with a width of ≈6 μm, and outwardly by a fringed verge, extending over a ≈5 μm wide ring, all around the spot. Moreover, several local defects with an elliptical shape decorate the transitional annular region (see panel (h)). The striking morphological features of this intermediate state of the surface is further analyzed by using AFM, optical and confocal microscopies and Raman imaging, as described later. It is worth noticing that the formation of different circular areas of modification during irradiation with a single or few fs pulses with a Gaussian beam profile in air was addressed earlier by Bonse *et al*.^20^ and Fuentes-Edfuf *et al*.^22^ and associated to regions of amorphization and re-crystallization. Anticipating our results, here we observe an outer halo of amorphous and crystalline silicon fringes surrounding a central, rippled crater and an intermediate annular region.

**Figure 2:**
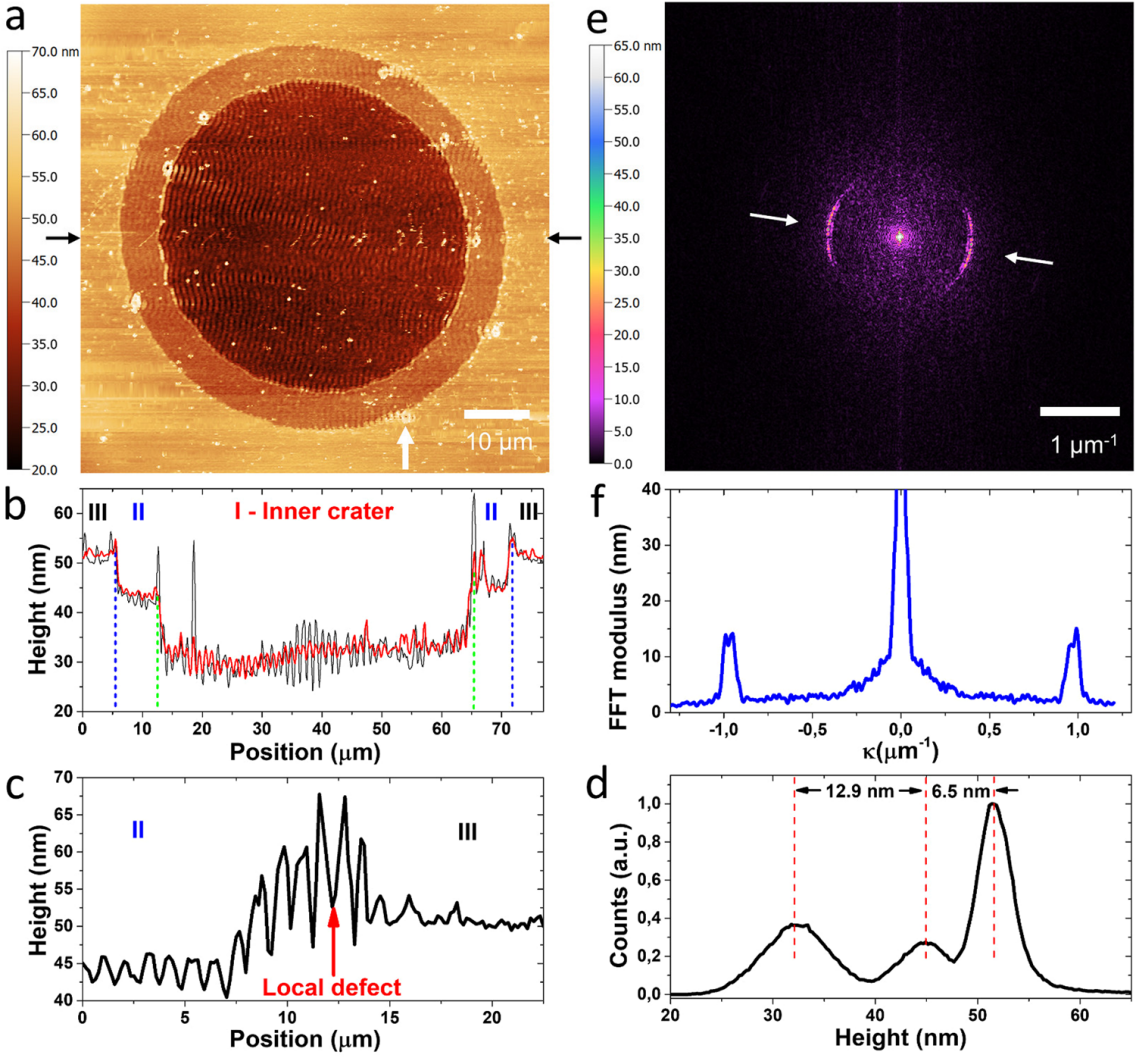
(**a**) AFM image of the silicon sample after irradiation with *N* = 4 laser pulses. (**b**) AFM topographic profiles along the horizontal direction indicated by the two black arrows in panel (a). The black and red curves show the profiles for a single line and averaged over multiple lines. The vertical dashed lines delimit the three region of the irradiated surface that are defined in the text.(**c**) Horizontal topographic profile around the local defect marked by the white arrow in panel (a). (**d**) Height distribution calculated from the whole AFM image. The three peaks correspond to the inner crater (I–32nm), the annular region (II–45 nm) and the unexposed surface (51 nm). The sensitivity of our instruments and the surface roughness do not allow us to distinguish between the unexposed surface and the fringed verge region (III). (**e**) Calculated 2D Fast Fourier Transform of the image shown in (a). (**f**) Cross-section of the 2D-FFT along the radial direction indicated by the white arrows in panel (e).

The topography of the silicon surface, measured by means of AFM, after irradiation with *N* = 4 pulses is illustrated in Fig. 2(a). Panel (b) shows the topographic profile along the horizontal diameter indicated by the black arrows in panel (a). The black and red curves in Fig. 2(b) refer to a single line and to an average over multiple lines. These yield further information about the depth of the various regions and the range of the ripples period and height. A profile around a local defect (marked with an arrow), at the edge between the annular region and the fringed verge, is also reported in panel (c).

The height profiles indicate that the internal crater is characterized by periodic ripples with a typical peak-to-valley distance of ≈10 nm and a period close to the laser wavelength. The intermediate annular region shows structures with a period similar to that of the ripples but a peak-to-valley distance of ≈3–4 nm. The various regions are separated by rims with a height of several nm. It is worth noticing that the external fringed verge region presents no significant topography.

Indeed line profiles show 1–2 nm features which are comparable with the surface roughness and with the sensitivity of our instrument. In order to estimate the depth of the various regions we report (panel (d)) the height distribution of the whole AFM image. This analysis indicates that the central, shallow crater and the surrounding annulus have average depths of ≈19.4 nm and ≈6.5 nm below the target surface. Therefore, an ablative regime is reached after few laser pulses in our experimental conditions.

Figure 2(e) reports the two-dimensional Fast Fourier Transform (2D-FFT) of the AFM image. This shows the characteristic sickle-shaped pattern typical of ripples predominantly oriented in direction perpendicular to the laser pulse polarization^5,23^. The slight tilt with respect to the horizontal direction of the 2D-FFT pattern is likely due to small uncertainty either in the alignment of the sample under the microscope or in the laser polarization direction. From a cross-section of the sickle-pattern along the laser polarization direction (panel (f)) we infer that the maximal amplitude of the FFT occurs at a wave-vector value κ = (0.98 ± 0.01) μm^−1^, indicating a ripple period Λ = 1/κ = (1.02 ± 0.01) μm. These ripples are mainly located inside the inner crater, whose topography also influences the optical images of the target surface inducing a spatial modulation of its reflectivity (see Supplementary Information, Sect. S1). It is worth noticing that other reflectivity features are present both in the fringed verge area surrounding the crater and around the elliptical defects seen in the SEM images. However, the AFM analysis suggests that these reflectivity modulations cannot be ascribed to significant topographic changes of the surface. Therefore, they likely result from a change of the local physical properties of the surface involving reflectivity and conductivity variations. Izawa *et al*. have shown that few laser pulses of fs laser irradiation can induce an ultrathin layer of amorphous Si (*a*-Si), depending on the wavelength and pulse fluence^24^. In fact, the formation of a thin *a*-Si layer is typically accompanied by a reflectivity increase at visible illumination wavelength^17,25^. Moreover, amorphous and crystalline phases of silicon laser processed surfaces present a significant difference in electrical conductivity^26^. To verify that the modulation observed in the SEM and optical images is due to localized phase changes resulting in an alternate disposition of amorphous (*a*-Si) and crystalline Si (*c*-Si) stripes, we carried out Raman spectroscopy and imaging of the irradiated sample surface.

**Figure 3:**
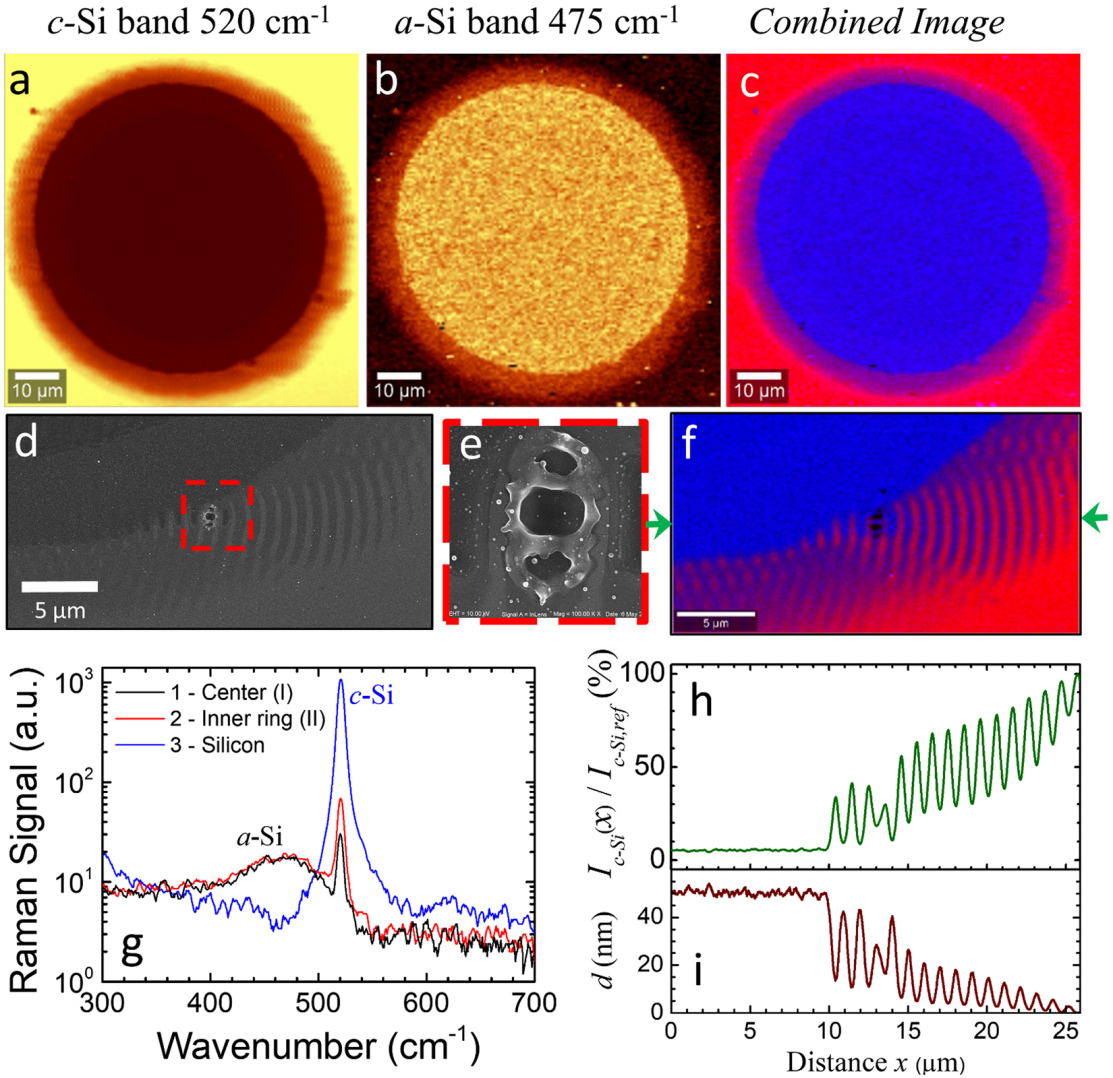
Raman images of the irradiated crater showing the integrated intensity of the Raman emission in the band of (**a**) *c*-Si centered at 520 cm^-1^ and (**b**) *a*-Si centered at 475 cm^-1^. (**c**) Combined Raman image of the silicon sample: red – *c*-Si; blue – *a*-Si. (**d**) IL-SEM image of the region around the local defects. (**e**) Magnified micrograph of the local defect registered in the area indicated as dashed boxes in panel (d). (**f**) 2D combined micro-Raman image of the area around the local defect: red – *c*-Si; blue – *a*-Si. (**g**) Raman spectra registered in different positions of the irradiated area from the center of the crater to the unexposed surface. (**h**) Profile of the Raman ratio *I*_*c-Si*_(*x*)/*I*_*c-Si,ref*_ along the horizontal line indicated by the two arrows in panel (d). (**i**) Depth profile of the *a-Si* layer as obtained by modeling the Raman ratio *I*_*c-Si*_(*x*)/*I*_*c-Si,ref*_ as a function of the amorphous overlayer thickness x.

Considering first the overall irradiated area, Fig. 3(a,b) report images of the sample surface at two specific Raman bands of *c-*Si and *a-*Si, respectively. These are obtained by integrating, at each point, the Raman spectrum in a narrow interval around the characteristic peaks of *c-*Si (520 cm^−1^) and *a-*Si (475 cm^−1^)^25,27^. The images indicate that the surface of the circular ablation crater (regions I and II) is prevalently composed by *a-*Si, meanwhile the surrounding fringed corona (region III) shows the coexistence of both Si phases. This is clearly illustrated in Fig. 3(c) that reports the 2D combined micro-Raman image of the sample and shows the coexistence of both *c-*Si and *a-*Si in the modified area of the Si sample. Panel (g) of Fig. 3 shows Raman spectra collected in various points (outside, inside and at the edge of the ablation crater). The unexposed area external to the ablation crater namely presents the sharp *c*-Si peak at 520 cm^−1^ (profile 3). The center of the crater (profile 1) features the Raman emission band of *a*-Si centered at 475 cm^−1^. The simultaneous presence of the *c*-Si peak in the same spectrum is related to the fact that the thickness of the *a*-Si layer is smaller than the microscope focal depth (1–2 µm), consequently a contribution of underlying *c*-Si is also recorded. The *c*-Si peak increases in the spectra recorded in the annular region of the crater (profile 2) due to the progressive thinning of the amorphous layer on the tail of the surface irradiated area.

Interestingly, the Raman analysis shows a halo at the periphery of the ablation crater where the crystalline and amorphous phases coexist, confirming that the extended fringed area surrounding the crater is due to the formation of gratings of alternate *a*-Si and *c*-Si stripes. Panel (d) of Fig. 3 report zoomed view of the sample surface in an area close to a local defect registered by IL-SEM, while panel (e) shows a magnified image of the defect addressing its peculiar morphology. The corresponding high resolution combined Raman image is shown in panel (f). In all these images, the defect is surrounded by a wavy surface pattern. The Raman view clearly demonstrates that this pattern is due to the formation of a grating of alternate *a*-Si and *c*-Si stripes.

Noteworthy, the observed pattern of phase grating is consistent with the energy spatial modulation predicted by Sipe-Drude model for Si at a low excitation level in the neighborhood of a point-like defect on a relatively flat surface^12^. This, in turn, suggests that the defect acts as a scattering center inducing a SSW and a local redistribution of the laser fluence, though interference with the low-energy tail of the incident Gaussian beam. The nature of the SSW is likely due to a SPP playing an important role in the case of silicon irradiated by ultrashort laser pulses^23,28^. The spatial modulation of the local energy absorption produces the striped succession of the Si phases registered in the microscopy images in adjacency of the defect. A consistency analysis, illustrated in Sect. 2 of Supplementary Information, supports a scenario in which a SSW in form of SPP likely generates the energy modulation leading to the formation of the *a*-Si/*c*-Si surface grating. This supports the idea that a modulation of the energy absorption occurs also in the surface regions irradiated by the lower fluence tail of the beam that do not undergo significant topographical changes and material removal. Moreover, the fact that striped patterns of the Si phase are present also in other region of the crater edge, besides those closely surrounding local defects, seems to herald the crater edge itself (e.g. rims and surface protrusions) as a more extended surface feature that induces the SSW. This, in turn, produces spatial gratings of the Si phases all around the nascent crater through interference between the SSW and the tail of the beam spot.

Panel (h) of Fig. 3 reports a cross-sectional profile of the *c*-Si Raman signal intensity, along the horizontal line identified by the two arrows, normalized to the value registered in an unaffected zone of the silicon sample. The spatial variation of the normalized intensity *I*_*c-Si*_(*x*)/*I*_*c-Si,ref*_ can be associated to the depth profile *d* of the *a*-Si overlayer along the cross-section. In fact, the presence of the *a*-Si overlayer on the *c*-Si target changes the intensity of the Raman signal because of both the attenuation of the exciting laser beam (at 488 nm) and the *c*-Si Raman signal (at 520 cm^−1^, wavelength ≈501 nm) and the interference effects due to the double-interface structure (see Supplementary Information, Sect. 3). This allows gaining direct information on the *a*-Si layer thickness in different locations, as reported in panel (i) of Fig. 3. One can observe that the inner ring region is characterized by an *a*-Si overlayer with a depth of ≈50 nm. The couple of fringes between the crater edge and the defect have depths varying from ≈40 nm to ≈10 nm. The *a*-Si/*c*-Si grating on the right of the local defect in Fig. 3(f) shows a decreasing trend as the distance from the defect increases with maximal depth going from ≈25 nm to ≈2 nm and interposed minimal depths ranging from ≈6 nm to ≈0 nm.

The formation of phase-change grating modifies the reflectivity of the silicon surface also in absence of significant topographic features resulting in an external halo characterized by reflectivity fringes departing from several localized defects as well as from the crater edge (see Optical and 2D confocal measurements in the Supplementary Information). A comparison of 2D confocal micrographs with the corresponding SEM and Raman images indicates that *a*-Si corresponds to the brighter areas in the confocal image and to the darker ones in the SEM image, in agreement with an increase of the *a*-Si reflectivity and a reduction of its electrical conductivity with respect to the pristine *c*-Si^25,26^.

The formation of regular stripes with alternate variation of the Si phase around the structured crater produced by a low number of pulses may play a potential role in the subsequent evolution of the surface structuring process. In fact, it might further mediate the modulation of the absorbed energy (feedback mechanism) in the peripheral areas of the laser beam spot and drive the successive development of the surface structures at larger number of laser pulses. Strikingly, such an effect has been seldom addressed earlier. We believe that this can be likely ascribed to the fact that direct laser surface structuring is typically performed in air at atmospheric pressure. Therefore, we carried out some target irradiation in air with the same laser pulses and contrasted the features of the formed surface structures with those obtained in vacuum, which is illustrated in the next section.

### Comparison between the surface structures generated in vacuum and at atmospheric pressure

**Figure 4:**
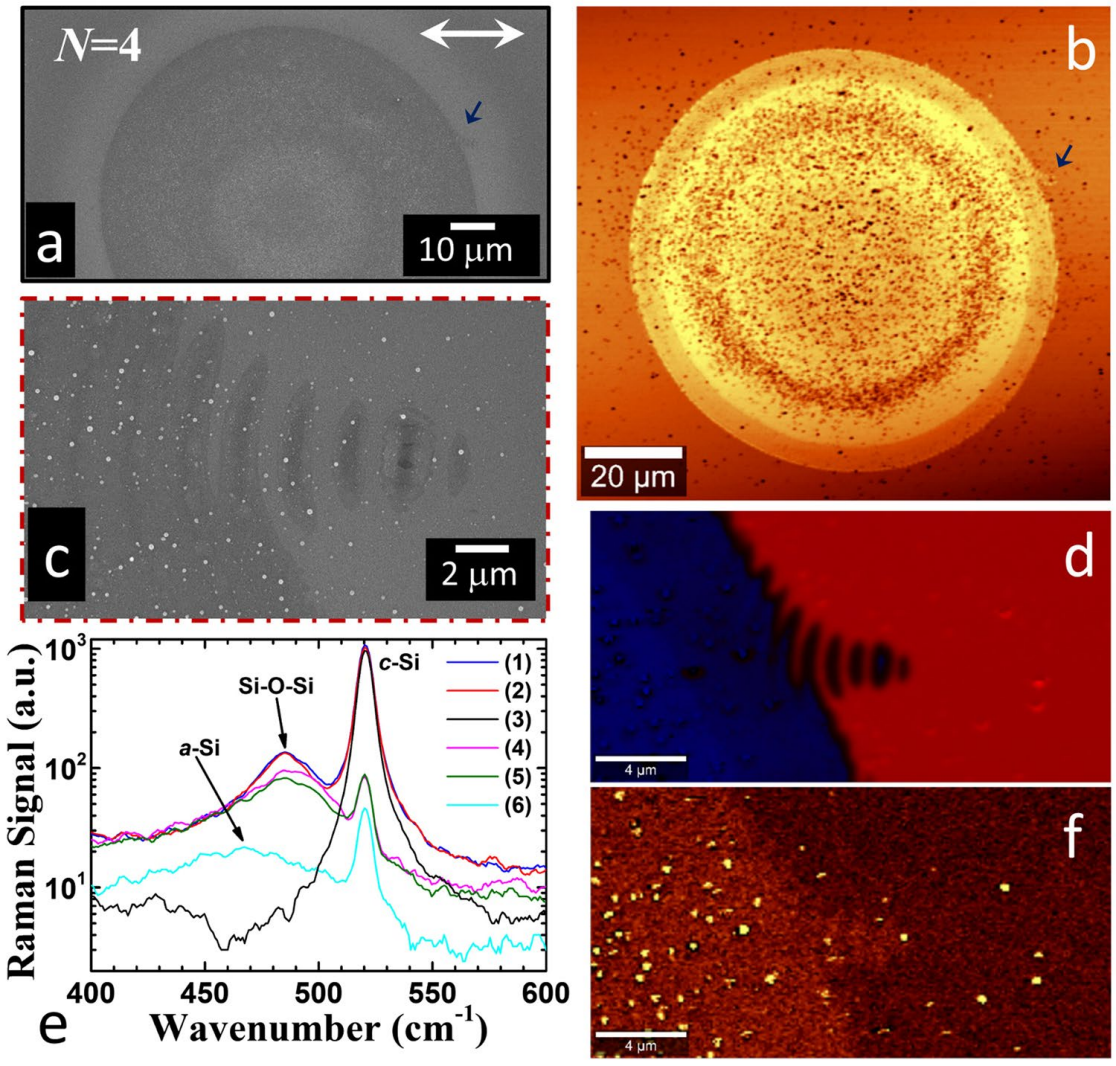
Microscopy analysis of the Si target surface after irradiation with *N* = 4 laser pulses in air. (**a**) SEM micrograph of the target surface. (**b**) Confocal image of the Si target surface. (**c**) Zoomed SEM view on the area indicated by the arrow in panels (a) and (b) showing a localized defect and fringes. (**d**) Combined Raman image of the area of the defect: red – *c*-Si; blue – *a*-Si. (**e**) Raman spectra registered in different positions of the irradiated area: (1) nanoparticle on *c*-Si; (2) nanoparticle on *c*-Si; (3) *c*-Si; (4) nanoparticle on *a*-Si; (5) nanoparticle on *a*-Si; (6) *a*-Si. (**f**) Raman image of the sample surface, around the defect, showing the Raman emission integrated in the Si-O-Si symmetric stretching band centered at 485 cm^−1^.

This comparison clarifies interesting issues related to laser surface structuring of Si, such as why the formation of a Si phase grating can be easily overlooked in air as well as the effects of the ambient pressure on ripples development. The results are summarized in Fig. 4, which reports SEM, confocal and Raman imaging results for a sample irradiated with *N* = 4 laser pulses in air, keeping fixed all the other experimental conditions. This can be directly compared with the corresponding case for vacuum reported above.

The target surface irradiated in air presents some marked differences with respect to that in vacuum, as indicated by SEM and confocal image of Fig. 4(a,b). In air, a nearly featureless amorphous spot is formed which is densely covered by nanoparticles. These nanoparticles extend over an area larger than the spot forming a halo on the almost unmodified, adjacent region around the shallow crater. The formation of an external layer of nanoparticles around a structured central region is a typical feature of direct fs laser surface structuring in air at higher number of laser pulses^1,29,30^. This is due to a strong backward flux of ablated material towards the target surface occurring during laser surface irradiation at atmospheric pressure^31^. Such a flux is typically composed by a large fraction of nanoparticles generated during fs laser ablation^32,33^.

In vacuum, such an effect is drastically reduced and the nanoparticles are very sparsely distributed over the target surface.

The confocal image of Fig. 4(b) also evidences a radial modulation of the reflectivity from the center of the spot that can be ascribed to a spatial variation of the amorphous layer thickness. A localized defect similar to the ones observed in vacuum can be identified close to the edge of the amorphous spot in both the SEM and confocal images of Fig. 4. SEM and combined Raman images of such a region, registered at higher spatial resolution, are shown in Fig. 4(c,d) respectively. They clearly indicate the formation of a wavy phase grating of *a*-Si and *c*-Si around the localized defect. Moreover, Raman imaging also confirms the prevalence of an amorphous silicon phase in the central area of the nascent crater^25^. Differently from vacuum, here the spatial extension of the *a*-Si/*c*-Si fringes around the defect is drastically reduced with the stripes extending for only few periods and with only one stripe at the left of the defect. This suggests that the large density of nanoparticles leads to an increased attenuation of the SSW and consequently to a reduction of its propagation length. Nonetheless, the extended frame of stripes with different Si phases around the crater is not formed in air, but a ring of nanoparticles is present instead. While this indicates that the formation of peculiar localized defects is also possible in air, the scarce presence of Si phase gratings surrounding the amorphous spot evidences the significant role played by the nanoparticles in mediating surface structure development at the early stage of the process.

**Figure 5:**
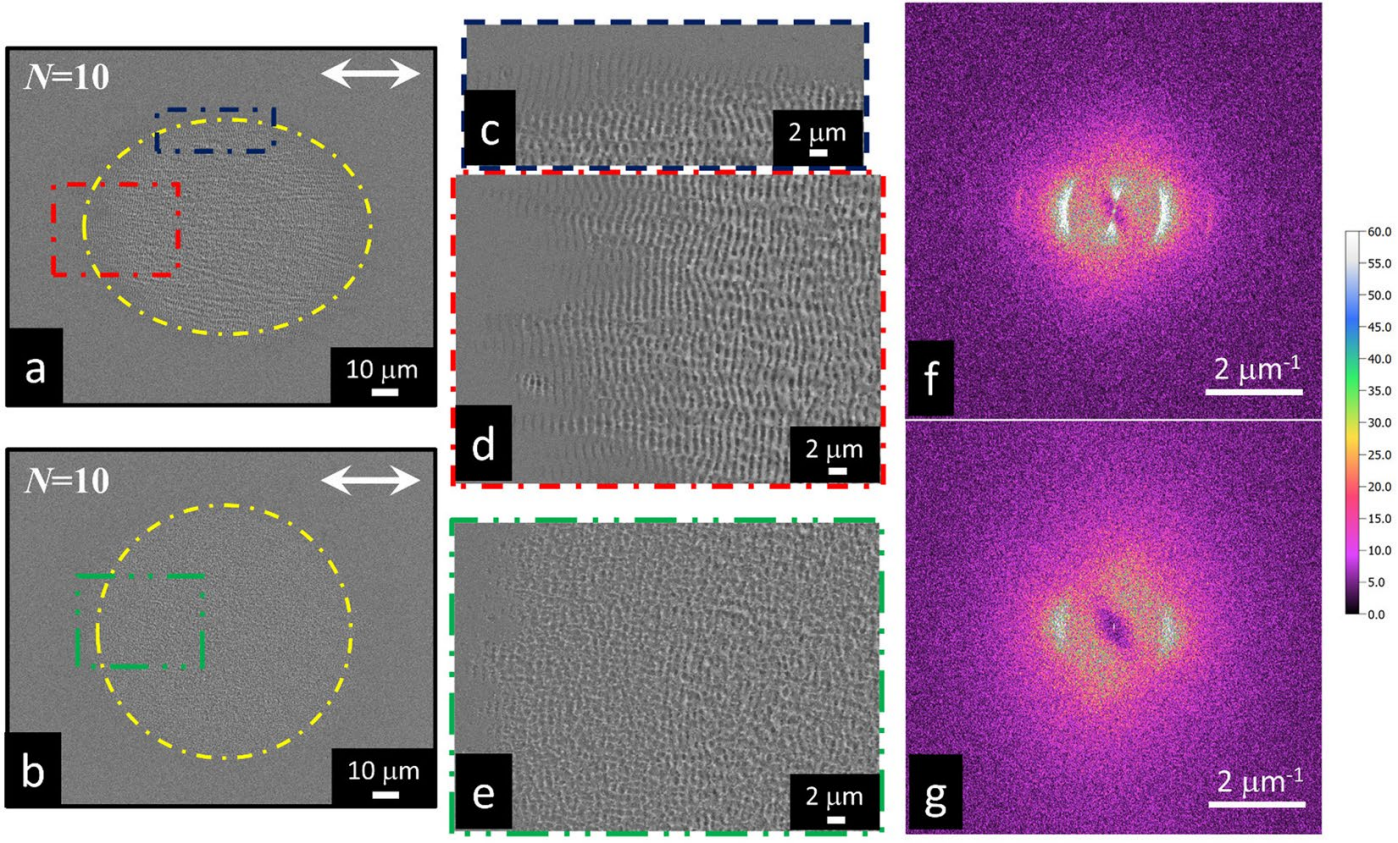
Panels (a) and (b) report SEM micrographs of the Si target surface after irradiation with *N* = 10 laser pulses in vacuum and in air, respectively. The yellow ellipse and circle in panels (a) and (b), respectively, approximately identify the region of the sample presenting surface structures. Panels (c) and (d) are zoomed views of the areas indicated by the boxes in panel (a) addressing the peculiar morphology of the surface structures at the upper and lateral edges of the elliptical structured region formed in vacuum conditions. Panel (e) is a magnified view of the area indicated by the box in panel (b) displaying the characteristic morphology of the sample surface in air. The double-headed arrow indicates the polarization of the incident laser pulses. Panels (f) and (g) are the calculated 2D-FFT of the SEM image in panel (a) and (b), displayed in the same FFT-units (nm) for comparison.

Interestingly, Raman spectra registered in the locations where nanoparticles are present show an additional peak at 485 cm^−1^, as evidenced in Fig. 4(e). This Raman emission is usually ascribed to Si-O-Si symmetric stretching^34^, and the reconstructed image at this specific Raman band reported in Fig. 5(f) clearly shows the enhanced contrast in the locations of the nanoparticles. The comparison between Raman spectra collected in areas covered by nanoparticles both on crystalline and amorphous silicon underlayers and in regions with a reduced coverage suggests that the signal is due to a partial oxidation of the nanoparticles generated during the first laser irradiation, while oxidation of the sample surface is negligible. Hence, oxidation process seems to result from a complex interaction of the generated nanoparticles with the surrounding air, before their back-deposition on the target surface.

We turn now to a comparison between the surface structures generated in vacuum and air, in the same experimental conditions, at a larger number of laser pulses, namely *N* = 10, when the typical ripples covering the laser irradiated surface starts forming. These represent the state of the surface that develops from the nascent structures analyzed at lower number of pulses and that subsequently drives the further progress of the surface texture through feedback mechanisms involved in laser surface structuring^3,28^.

Panels (a) and (b) of Fig. 5 report two SEM images of the Si target surface after irradiation in vacuum and in air, respectively. Moreover, panels (c), (d) and (e) of Fig. 5 show zoomed views of the structured sample surface in such experimental conditions. Interestingly, the structured surface region presents a different shape in the two cases. In vacuum (panel (a)), the structured region can be roughly delimited within an ellipse preferentially elongated in the direction of the laser polarization and whose major and minor axes are ≈100 μm and ≈80 μm, respectively. In air (panel (b)), instead, the surface structures occupy a nearly circular area with a diameter of ≈95 μm. Moreover, zoomed views of the sample surface also indicate that in vacuum (panels (c) and (d)) the ripples are already formed after *N* = 10 laser pulses. Instead, the morphological features of the surface structures formed in air by the same number of laser pulses (panel e) have a still rather random character and well developed ripples are found only at larger number of pulses (i.e. *N* ≈ 50, not shown), in our experimental conditions.

Panel (c) and (d) show that the elliptical shape of the structured area in vacuum results from the creation of a jagged surface texture with many rippled, arrowhead protrusions pointing along the direction of the laser polarization and extending for several microns outwards the main spot. This texture is possibly promoted by a feedback effect involving the Si phase gratings formed at lower number of pulses and subsequently reinforced by the progressive creation of oriented ripples.

In Fig. 5, the Fourier analysis of the SEM images in vacuum (panel (f)) and in air (panel (g)) quantifies the differences in periodicity and regularity of the surface structures formed in vacuum and air, in the same laser irradiation conditions. In vacuum the 2D-FFT presents a bright sickle-shaped pattern with narrow maxima along the horizontal cross-section profile, indicating a well-defined periodicity of the ripples. Interestingly, two narrow spots emerge in the vertical direction at a low wavenumber of about 0.35 μm^−1^. Those spatial frequencies represent undulation of the surface in direction orthogonal to that of the incident polarization and with a period three times larger than that of the incident radiation. In air, the 2D-FFT shows a wider and less intense range of spatial frequencies (cloud) roughly centered around the two fundamental wave vectors observed in vacuum and with broad maxima along the cross-section profile. Two vertical clouds are present too, but the overall image approaches a ring-shaped pattern of spatial frequencies indicating a lower degree of ordering of the surface structures. Therefore, the FFT analysis demonstrates that LIPSS fabricated in vacuum exhibit a higher degree of regularity than those formed in air in the same experimental conditions. Such an observation further support the idea that the strong nanoparticle decoration of the surface occurring in air has important, direct influences on the creation and evolution of the surface structures.

In conclusion, we have carried out a correlative imaging analysis of the nascent surface structures that are produced by fs laser irradiation on silicon in vacuum with low number of laser pulses. This experimental condition, seldom investigated, limits back-deposition of nanoparticles and allows gaining further intriguing details of the laser structuring process. The surface structures have been characterized with several microscopy techniques (optical, atomic force, electron and Raman). Our multi-imaging approach evidences that, besides the typical topographic ripples present in a shallow crater, phase-change gratings consisting of alternating amorphous and crystalline periodic stripes, with no material removal, form at its periphery. The source of these phase gratings has been identified in two kinds of surface defects: the edge of the nascent ablation crater and peculiar lobe-shaped structures generated in the very first laser pulses. Our analysis convincingly addresses the role of these defects on emerging ordered structures at the first stage of the process that subsequently influence the successive evolution dynamics of the laser-irradiated surface. Finally, the comparison with results obtained at atmospheric air pressure, in the same experimental conditions, have confirmed the important role played by nanoparticles decorating the samples surface in this case and the merits of vacuum processing.

## Methods

The laser source is a Nd:glass laser system that delivers linearly polarized pulses with a Gaussian spatial profile at a repetition rate of 33 Hz. The pulse duration is ≈850 fs and the wavelength is 1055 nm. The target is a commercial, intrinsic (resistivity >200 Ω cm) (100) oriented crystalline silicon plate. The target is mounted on a XY-translation stage perpendicular to the laser beam direction and located in a vacuum chamber at a base pressure of ≈10^−5^ mbar. The laser beam is focused on the target by a plano-convex lens to a spot radius *w*_0_ = (130 ± 5) µm, determined by measuring the energy variation of the ablation crater dimensions^35^. The experiment is carried out in static conditions by applying *N* consecutive laser shots to the same spot on the target by exploiting an electromechanical shutter.

The sample is characterized *ex-situ* by different microscopy techniques. Scanning electron microscopy (SEM) of the irradiated surface is carried out by using a field-emission scanning electron microscope (FESEM, model Zeiss ∑igma). The FESEM is equipped with two detectors for secondary electrons (SE): a conventional Everhart-Thornley (ET) detector and an In-Lens (IL) detector. The IL detector is located inside the electron column of the microscope and arranged rotationally symmetric around its axis. It collects with high efficiency the SE that are generated by the incoming electron beam as electrons enter the surface, providing images more sensitive to the surface properties. The ET detector images are based on SE that have returned to the surface after several inelastic scattering events in the sample. As the SE come from layers underneath the surface their image is dominated by morphological features mainly related to topographical properties. Sample topography is measured by means of an Atomic Force Microscope (AFM – Witec Alpha 300 RAS) working in tapping mode with non-contact cantilevers (resonance frequency of 280 kHz and spring constants of k = 42 N/m). Raman micro-analysis of the sample is carried out through a scanning confocal optical microscope using a 488 nm laser excitation wavelength (5 mW power) and a high numerical aperture microscope objective (50x magnification, 0.75 NA). The backscattered signal is filtered by two high quality long-pass filters to get rid of the pump beam. It is collected by an optical fiber (50 µm core which acts as confocal pinhole) and sent to a 300 mm focal length spectrometer equipped with a thermo-electric cooled, back-illuminated CCD detector^36^. Images of the sample surface at a specific Raman band are reconstructed by acquiring a full spectrum at each pixel (5 ms integration time) of the optical scanning and then integrating the signal in the corresponding spectral range. Confocal images are registered with the same optical setup of the Raman spectroscopy, by removing one of the two long-pass filter, in order to collect the pump beam back-reflected at the sample surface. Spatial resolution of our confocal images is about 350 nm. Optical micrographs of the sample are also collected by means of an optical microscope working in reflection with bright field illumination.

## Electronic supplementary material

Supplementary Information
