# Supplementary material for: Analysis of nascent silicon phase-change gratings induced by femtosecond laser irradiation in vacuum
Source: Sci Rep. 2018 Aug 21;8:12498. doi: 10.1038/s41598-018-30269-0 (PMC6104067; doi:10.1038/s41598-018-30269-0)
Supplement: Supplementary file 1 — Supplementary Information [file 41598_2018_30269_MOESM1_ESM.pdf]

Supplementary Information

Analysis of nascent silicon phase-change gratings induced by femtosecond laser irradiation in vacuum

Felice Gesuele[1], Jijil JJ Nivas[1,2], Rosalba Fittipaldi[3], Carlo Altucci[1], Riccardo Bruzzese[1,2], Pasqualino Maddalena[1], Salvatore Amoruso[1,2]

1-Dipartimento di Fisica "Ettore Pancini", Università di Napoli Federico II, Complesso Universitario di Monte S. Angelo, Via Cintia, I-80126 Napoli, Italy.

2-CNR-SPIN UOS Napoli, Complesso Universitario di Monte S. Angelo, Via Cintia, I-80126 Napoli, Italy.

3-CNR-SPIN, UOS Salerno, Via Giovanni Paolo II 132, I-84084 Fisciano, Italy.

Abstract

This document provides supplementary information to "Analysis of nascent silicon phase-change gratings induced by femtosecond laser irradiation in vacuum". We report on the surface scattered wave consistency analysis, the modeling of a double interface for the rationalization of Raman and reflectivity cross-sectional profiles obtained by micro-Raman and confocal imaging analyses. Additional experimental results are also included.

S1. Optical image of the sample surface.

Figure S1 shows an optical image of the sample after irradiation with N=4 laser pulses at an energy E_0=140 μJ, in high vacuum. One can observe that the morphology of the ripples located inside the inner crater also influences the optical images of the target surface inducing a spatial modulation of its reflectivity. Interestingly, reflectivity modulations are also observed outside the shallow ablation crater, namely in the fringed verge area around the crater and in the area of the gratings departing from the elliptical defects seen in the SEM image. The AFM analysis discussed in the main text indicates that these regions are not characterized by significant topographic changes of the surface, thus suggesting that the reflectivity changes in such areas can be ascribed to a change of the local physical properties of the surface, as confirmed by micro-Raman imaging.

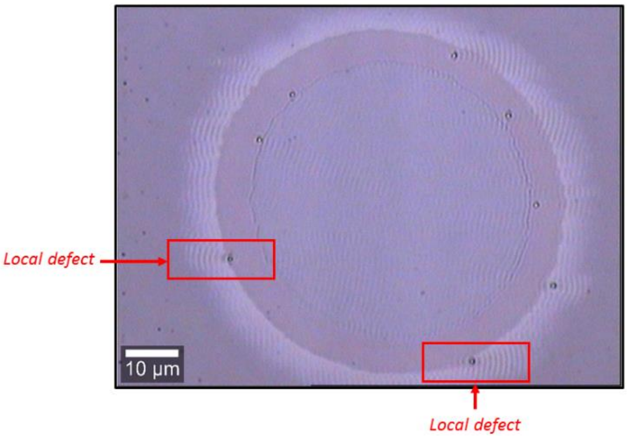

Figure S1. Optical image of the sample surface after an irradiation sequence of N=4 laser pulses at an energy E_0=140 μJ, in high vacuum.

S2. Consistency analysis on surface scattered wave.

Here we report a consistency analysis about the formation of the amorphous-crystalline fringes through the generation of a surface scattered wave (SSW). Under ultrashort laser pulses irradiation, the complex refractive index of silicon can be noticeably modified as a consequence of the high

density of generated electrons. In such excited conditions, surface roughness and defects can act as scattering centers for the formation of SSW, as e.g. Surface Plasmon Polariton (SPP)[1–3], that interfering with the incident laser beam give rise to a modulation of the absorbed energy. This scenario is typically associated to the formation of the morphological ripples generated during fs laser irradiation of solid targets. In particular Huang et al. proposed SPP as a mechanism explaining the typical decrease of ripples period with number of laser pulses *N* through a grating-assisted influence on the ripples period[2]. However, a similar effect has been rationalized on a more general SSW approach by Zhang et al.[4], thus indicating SSW as a general aspect of the feedback mechanisms involved in ripples generation by fs laser irradiation, which is further enhanced for SPP-active targets as silicon under ultrashort laser pulses[3]. Hereafter we try to support a scenario where a SSW in form of SPP can generate an energy modulation leading to the formation of the *a*-Si/*c*-Si surface grating. The relative dielectric constant of the excited silicon surface ε_{ex}, at the laser wavelength ω_L, can be expressed as[2,5,6]:

$$\varepsilon_{ex} = \varepsilon_{ex,1} + i\,\varepsilon_{ex,2} = \varepsilon_0(\omega_L) - \frac{\omega_p^2}{\omega_L^2 + i\,\omega_L \Gamma} \qquad \text{(S.01)}$$

where $\varepsilon_0(\omega_L) = 12.5+0.001i$ is the relative dielectric constant of silicon[7] in normal state at the laser wavelength ω_L ($=1.787\times10^{15}$ s^{-1}), $\omega_p = \sqrt{\frac{e^2 N_e}{\varepsilon_0 m_{eff}}}$ the plasma frequency (e is the electron charge, N_e the electron density, ε_0 the dielectric permittivity of vacuum and m_{eff} the effective optical electron mass; $m_{eff} \approx 0.18\, m_e$, m_e being the electron mass) and Γ the electron collision frequency. From Eq. (1), one can derive for the real and imaginary part of the dielectric constant ε_{ex}:

$$\varepsilon_{ex,1} = \varepsilon_0(\omega_{las}) - \frac{\omega_p^2}{\omega_L^2+\Gamma^2}; \qquad \varepsilon_{ex,2} = \frac{\omega_p^2 \Gamma}{\omega_L\,(\omega_L^2+\Gamma^2)} \qquad \text{(S.02)}$$

Assuming that $\varepsilon_{ex,2} <| \varepsilon_{ex,1}|$, the SPP and the incident laser interfere forming a fringe pattern with a period

$$\Lambda = \lambda_L Re\left[\sqrt{\frac{\varepsilon_d + \epsilon_{ex,1}}{\varepsilon_d\ \epsilon_{ex,1}}}\right] \tag{S.03}$$

while the SPP propagation length L_s along the surface is given by:

$$L_s = \frac{\lambda_L}{2\pi}\left(\frac{\varepsilon_d + \epsilon_{ex,1}}{\varepsilon_d\ \epsilon_{ex,1}}\right)^{\frac{3}{2}} \frac{\varepsilon_{ex,1}^2}{\varepsilon_{ex,2}} \tag{S.04}$$

where ε_d is the relative dielectric constant of the dielectric material (i.e. $\varepsilon_{d}=1.0$ in our case)[2,5,6]. Our findings show a period of the *a*-Si/*c*-Si surface fringes $\Lambda \approx 1.0$ μm, from which one can estimate a value of $\varepsilon_{ex,1} \approx -9.8$, which indicates a condition of the irradiated surface consistent with a metallic state of the excited Si ($\varepsilon_{ex,1} < -1$) that can support SPP. For an electron collision frequency $\Gamma = 1.5\times10^{14}$ s characteristics of Si in condition of ripples formation[3], from Eq. (2a) one can estimate a plasma frequency $\omega_p \approx 8.5\times10^{15}$ s^{-1}, also fulfilling the condition $\omega_L < \omega_p$ for SPP generation. Then, Eq. (2b) yields $\varepsilon_{ex,2} \approx 1.8$ and a corresponding SPP propagation length $L_s \approx 7$ μm, which is consistent with the observed extension of the *a*-Si/*c*-Si fringes of about 10-15 μm.

S3. Double-interface model for the analysis of Raman spatial profiles.

In Figures 3(f) we have shown a 2D combined micro-Raman image of the area around the local defect from which a cross-sectional profile of the Raman ratio $I_{c\text{-}Si}(x)/I_{c\text{-}Si,ref}$ is obtained as shown in Fig. 3(h). These data have been elaborated to derive a profile of the *a-Si* layer thickness, *d*, by considering as a schematic model an *air*/*a-Si*/*c-Si* double interface system with the *a-Si* layer

separating two semi-infinite slabs of air and *c-Si*. On the basis of the Raman characterization that does not show any sizeable contribution coming from silicon oxide, we have neglected it in our approach. The model, sketched in Fig. S2, considers the influence of the partially absorbing and reflecting *a*-Si layer on the intensity of the Raman signal at the 520 cm^{-1} *c*-Si band (wavelength ≈501 nm) due to both attenuation of the exciting and Raman-shifted photons at 488 nm and 500.7 nm, respectively, and the interference effects of the double-interface structure. Moreover, the analysis is carried out for normal incidence angle.

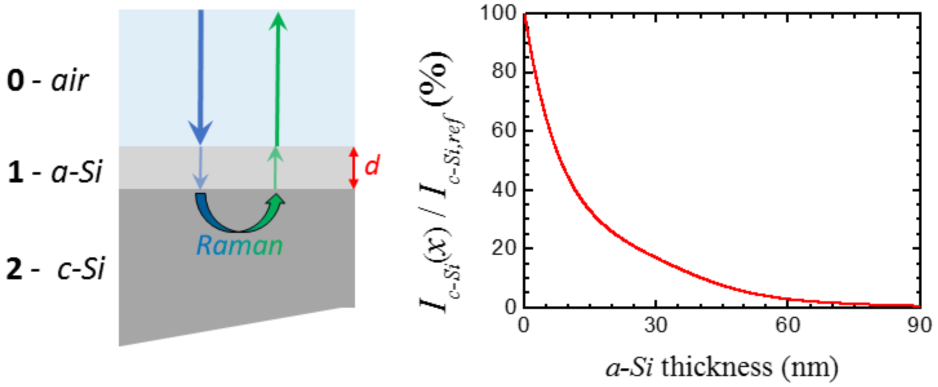

Figure S2. (left) Schematic of the air/a-Si/c-Si double interface system with the *a*-Si layer of thickness *d* separating two semi-infinite slabs of air and *c*-Si. (b) variation of the Raman signal ratio $I_{c\text{-}Si}(x)/I_{c\text{-}Si,ref}$ as a function of the *a-Si* overlayer thickness *d*.

As for the exciting beam, these effects are described by evaluating the transmissivity of the double-interface by using the complex index of refraction of *a-Si* and *c-Si* at λ_{ex}=488 nm, and the intensity reaching the underlying *c-Si* layer is given by:

$$I_{ex} = T_{ex}(d) I_{inc} \quad \text{(S.05)}$$

with

$$T_{ex}(d) = \left| \frac{n_{2,ex}}{n_{0,ex}} \frac{t^{ex}_{0\to 1}\, t^{ex}_{1\to 2}\, e^{ik_{1,ex}d}}{1 - r^{ex}_{0\leftarrow 1} r^{ex}_{1\leftarrow 2} e^{2ik_{1,ex}d}} \right|^2 \quad \text{(S.06)}$$

where n_0 and $n_{2,ex}$ are the complex refractive indices of air and *c-Si* and $t_{0\to1}^{ex}$, $t_{1\to2}^{ex}$, $r_{0\leftarrow1}^{ex}$, $r_{1\leftarrow2}^{ex}$ the single-boundary complex Fresnel coefficients for the passage at the interface *n, l (n and l =0,1,2, with n≠ l)* for transmission (*t*) and reflection (*r*) at the excitation wavelength[8]. In Eq. (S.06), $k_{1,ex} = 2\pi\, n_{1,ex}/\lambda_{ex}$ is the complex wave-vector modulus, with $n_{1,ex}$ the complex refractive index of *a-Si* at the excitation wavelength λ_{ex}. The arrow in the subscripts indicate the direction of propagation for the given contribution, taking into account that the forward-traveling wave from layer *0* to *2* in the middle region *1* arises from both a transmission of the incident wave and a reflection of the backward-traveling wave in the middle region that get reflected at the first interface[8].

Layer	***Refractive index***
air - 0	$n_{0,ex} = n_{0,R} = 1.00$
a-Si - 1	$n_{1,ex} = 4.478 + 1.186\, i$ - $n_{1,R} = 4.470 + 1.120\, i$
c-Si - 2	$n_{2,ex} = 4.367 + 0.079\, i$ - $n_{1,R} = 4.298 + 0.073\, i$

Table I: Values of the complex refractive indices used in the modeling of the optical properties of the samples for Raman and Confocal imaging.

The intensity I_R of the light generated into layer 2 by Raman scattering is given by:

$$I_R = \varepsilon_R I_{ex} \tag{S.07}$$

where ε_R is an efficiency factor of the Raman scattering process. Then, the backscattered light at the shifted Raman wavelength travels the layer and is eventually detected as I_{det}:

$$I_{det}(d) = T_R(d) I_R \tag{S.08}$$

with

$$T_R(d) = \left| \frac{n_{0,R}}{n_{2,R}} \frac{t^R_{2\to1}\, t^R_{1\to0}\, e^{ik_{1,R}d}}{1 - r^R_{2\leftarrow1} r^R_{1\leftarrow0} e^{2ik_{1,R}d}} \right|^2 \quad \text{(S.09)}$$

where $n_{0,R}$ and $n_{2,R}$ are the complex refractive indices of air and *c-Si*, and $t^R_{2\to1}$, $t^R_{1\to0}$, $r^R_{2\leftarrow1}$, $r^R_{1\leftarrow0}$ the single-boundary complex Fresnel coefficients for the passage at the *n*, *l* interface *(n and l =0,1,2, with n≠ l)* for transmission (*t*) and reflection (*r*) at the Raman-shifted wavelength. In Eq. (S.09), $k_{1,R} = 2\pi\, n_{1,R}/\lambda_R$ is the complex wave-vector modulus, with $n_{1,R}$ the complex refractive index of *a-Si* at the Raman-shifted wavelength λ_R=500.7 nm. Hence, the registered signal is:

$$I_{det}(d) = \varepsilon_R T_R(d) T_{ex}(d) I_{inc} \quad \text{(S.10)}$$

If one normalizes the detected signal at the reference *c-Si* one registered in an unmodified region, I_{det} (*d*=0), one eventually gets:

$$\frac{I_{c-Si}(d)}{I_{c-Si,ref}} = \frac{T_R(d) T_{ex}(d)}{T_R(0) T_{ex}(0)} \quad \text{(S.11)}$$

The variation of the Raman signal ratio $I_{c\text{-}Si}(x)/I_{c\text{-}Si,ref}$ as a function of the *a-Si* overlayer thickness *d* is reported in the right panel of Fig. S2, and it has been used to derive the *a-Si* thickness profile reported in Fig. 3(i). The values of the complex refractive indices at the two wavelengths have been taken by Palik[9] and summarized in Table I.

S4. Confocal microscopy and double-interface model for the analysis of reflectivity spatial profiles.

Fig. S3(a) reports a confocal image of the Si sample surface for *N*=4 laser pulses. Besides the rippled area in the internal region of the crater showing a variation of reflectivity likely induced by the topography, the external region shows an extended halo with reflectivity fringes departing from several localized defects as well as from the crater edge. The comparison of 2D confocal micrographs with the corresponding SEM and Raman images indicates that *a*-Si corresponds to

the brighter areas in the confocal image and to the darker ones in the SEM image, in agreement with an increase of the *a*-Si reflectivity and a reduction of its electrical conductivity with respect to the pristine *c*-Si.[10,11]

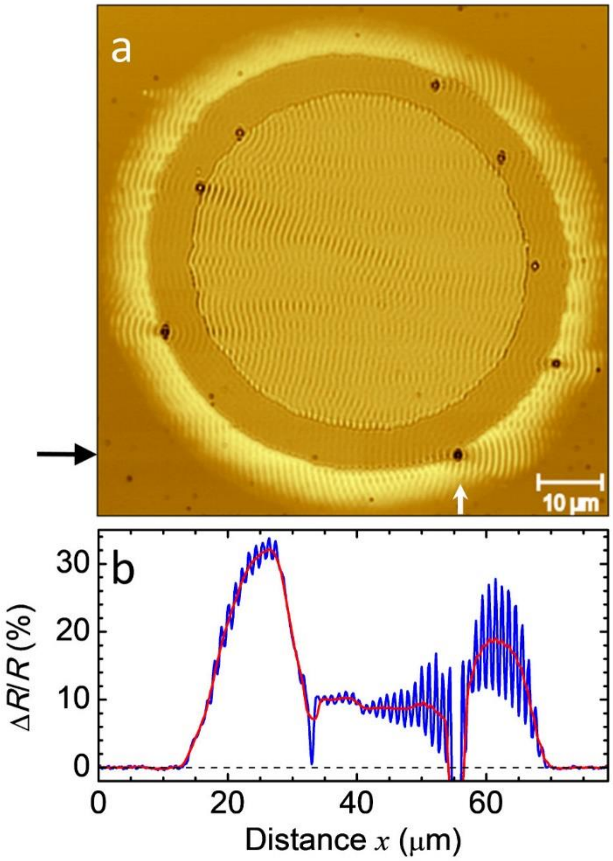

Figure S3. (a) Confocal image of the Si target surface after irradiation with N=4 laser pulses (a vertical white arrow indicates the location of the defect reported in Fig. 3). **(b)** Profile of the surface reflectivity $\Delta R/R$ (blue curve) and average value (red curve) along the line indicated by the horizontal black arrow in panel (a).

Fig. S3(b) reports a cross-sectional profile of the relative variation of the reflectivity, $\Delta R/R$, along a horizontal line identified by the arrow in Fig. S3(a). $\Delta R/R$ has been obtained by subtracting from the local reflectivity signal, $R(x)$, the asymptotic reference value, R_{ref}, registered at larger distance from defects, where the reflectivity becomes constant, and then normalizing it to R_{ref}, i.e.

$\Delta R/R=(R(x)-R_{ref})/R_{ref}$. The reflectivity profile of Fig. S3(b) shows a series of fringes associated to the progressive passage from *a*-Si to *c*-Si superimposed over an average variation of the local reflectivity (red curve). Moreover, in Fig. S3(b) two localized defects and the crater edge can be identified by the two sudden dips, at $x\approx55$ μm and $x\approx33$ μm, and a shoulder, at $x\approx31$ μm, respectively. Moving outwards the spot, the reflectivity variation reaches a maximum which is then followed by a progressive decay towards the reference value corresponding to *c*-Si.

The reflectivity of fs laser irradiated silicon was analyzed earlier by considering a thin film optical model to address the influence of the *a*-Si layer thickness for modified regions of the Si surface extending over several μm[12,13]. A similar analysis has been carried out to rationalize the spatial profiles of the observed reflectivity registered by confocal microscopy, as e.g. in Fig. S3.

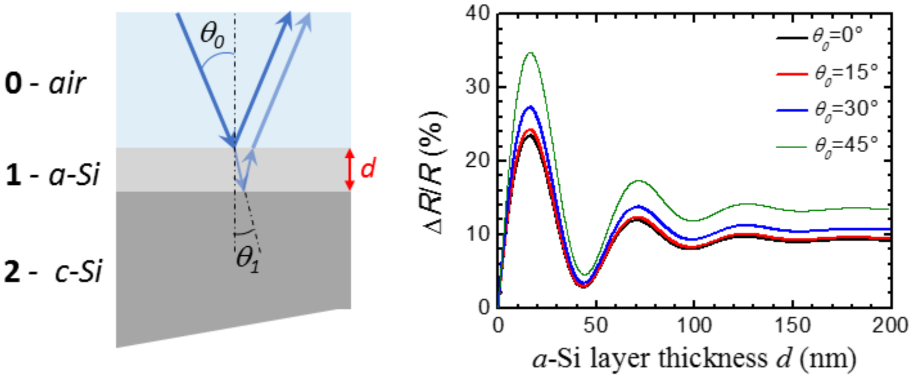

Figure S4. (left) Schematic of the *air*/*a-Si*/*c-Si* double interface system with the *a*-Si layer of thickness *d* separating two semi-infinite slabs of *air* and *c-Si*. (right) Variation of the relative reflectivity ratio $\Delta R/R$ as a function of the *a-Si* layer thickness for p-polarized light at 488 nm, for different values of the incident angle θ_0.

Considering the complex refractive indices of the various layers at the confocal laser wavelength λ_{ex}, the variation of the reflectivity ratio $\frac{\Delta R}{R}=\frac{R(d)-R_{ref}}{R_{ref}}$, where R_{ref} corresponds to the reflectivity

of the silicon target registered in an unmodified area, i.e. $R_{ref} = R(d{=}0)$, can be estimated. The model is sketched in Fig. S4 and p-polarized light is considered, as in our experimental conditions. The reflectivity of the double-interface is given by[8]:

$$R(d) = \left| r_{0\to1} + \frac{t_{0\to1} r_{1\to2} t_{0\leftarrow1} e^{2ik_{1,ex}d\cos(\theta_1)}}{1 - r_{0\leftarrow1} r_{1\to2} e^{2ik_{1,ex}d\cos(\theta_1)}} \right|^2 \quad \text{(S.11)}$$

where $r_{n\leftrightarrow l}$ and $t_{n\leftrightarrow l}$ are the single-boundary complex Fresnel coefficients for the passage at the interface *n, l (n and l =0,1,2, with n≠l)* in the propagation direction indicated by the arrow and θ_1 is the angle of propagation in layer 1. Hence, the reflectivity ratio $\frac{\Delta R}{R}$ shown in Fig. S4, for various angle of incidence θ_0, is obtained. One can observe a damped oscillating behavior of the reflectivity ratio due to both absorption and interference effects of the double-interface. By considering the average reflectivity values in Fig. S3(b) for x >55 μm, a maximum *a*-Si depth of ≈24 nm can be inferred for the fringes produced around the intense defect. As for fringes produced by the crater edge (x <30 μm), $\Delta R/R$ reaches average values larger than that expected for normal incidence of the probing beam of the confocal microscope. In first instance, a normal incidence can be considered for the confocal microscope measurements; however, the non-monotonic character of the relative reflectivity ratio dependence on the *a-Si* layer thickness makes such approach less reliable than the micro-Raman one. Experimental results (shown in Fig. S3(b)) indicate a maximum value of the average $\Delta R/R$ in the fringe pattern at the right of the defect of ≈20%, which might correspond to a maximal depth of ≈24 nm if the decreasing part after first maximum is considered in Fig. S4. This value is consistent with the estimate obtained by micro-Raman imaging, however sizeable oscillations around the average values of the reflectivity ratio are observed experimentally, in agreement with previous reports concerning larger surface area modification of silicon with ultrashort laser pulses[10]. There can be various reasons for such a

behavior. First, deviations from the normal incidence of the confocal probing radiation, related to the high numerical aperture of the objective, might lead to a larger variation of the reflectivity ratio, as predicted by the model at increasing incident angles in Fig. S.4. Second, differences might also arise from the fact that a double-interface air/*a*-Si/*c*-Si system with perfectly parallel surfaces exploited in the model cannot completely describe subtleties coming from undulations and reduced sharpness of the real structure in the description of the surface reflectivity. In the real case both interfaces should present undulations and reduced sharpness. However, the fringes can be associated to a thickness modulation of the *a*-Si overlayer around its average value, which is larger for fringes produced by the SSW induced at the local defect than for the crater edge. This can be likely ascribed to a superposition of several scattered wavelets, each with a different phase, interfering with the Gaussian spot in the region around the extended crater edge, while a more localized source of SSW with well-defined phase can be considered for the punctual defect. Further analyses should be necessary to completely clarify such an aspect.

References

1. Bonse, J., Hoehm, S., Kirner, S. V., Rosenfeld, A. & Krueger, J. Laser-Induced Periodic Surface Structures-A Scientific Evergreen. *IEEE J. Sel. Top. Quantum Electron.* **23,** 109–123 (2017).

2. Huang, M., Zhao, F., Cheng, Y., Xu, N. & Xu, Z. Origin of laser-induced near-subwavelength ripples: Interference between surface plasmons and incident laser. *ACS Nano* **3,** 4062–4070 (2009).

3. Derrien, T. J. Y., Itina, T. E., Torres, R., Sarnet, T. & Sentis, M. Possible surface plasmon polariton excitation under femtosecond laser irradiation of silicon. *J. Appl. Phys.* **114,** 083104 (2013).

4. Zhang, H. *et al.* Coherence in ultrafast laser-induced periodic surface structures. *Phys. Rev. B* **92,** 174109 (2015).

5. Raether, H. *Surface Plasmons on Smooth and Rough Surfaces and on Gratings*. **111,** (Springer Berlin Heidelberg, 1988).

6. Maier, S. A. *Plasmonics: Fundamentals and applications*. *Plasmonics: Fundamentals and Applications* (Springer US, 2007). doi:10.1007/0-387-37825-1

7. *Optical properties of selected elements - CRC handbook of chemistry and physics*. (CRC Press, 2005).

8. Peatross, J. & Ware, M. *Physics of Light and Optics*. (Brigham Young University, 2015).

9. Palik, E. *Handbook of Optical Constants of Solids*. (Academic Press, 1997).

10. Bonse, J., Brzezinka, K. W. & Meixner, A. J. Modifying single-crystalline silicon by femtosecond laser pulses: An analysis by micro Raman spectroscopy, scanning laser microscopy and atomic force microscopy. *Appl. Surf. Sci.* **221,** 215–230 (2004).

11. Alpuim, P., Cerqueira, M. F., Iglesias, V., Machado, G. & Borme, J. Laser patterning of amorphous silicon thin films deposited on flexible and rigid substrates. *Phys. status solidi* **213,** 1717–1727 (2016).

12. Bonse, J., Rosenfeld, A. & Krüger, J. On the role of surface plasmon polaritons in the

formation of laser-induced periodic surface structures upon irradiation of silicon by femtosecond-laser pulses. *J. Appl. Phys.* **106,** 104910 (2009).

13. Fuentes-Edfuf, Y. *et al.* Fabrication of amorphous micro-ring arrays in crystalline silicon using ultrashort laser pulses. *Appl. Phys. Lett.* **110,** 211602 (2017).
